# Activity Increase Despite Arthritis (AÏDA): design of a Phase II randomised controlled trial evaluating an active management booklet for hip and knee osteoarthritis [ISRCTN24554946]

**DOI:** 10.1186/1471-2296-10-62

**Published:** 2009-09-04

**Authors:** Nefyn H Williams, Elvis Amoakwa, Kim Burton, Maggie Hendry, John Belcher, Ruth Lewis, Kerenza Hood, Jeremy Jones, Paul Bennett, Rhiannon T Edwards, Richard D Neal, Glynne Andrew, Clare Wilkinson

**Affiliations:** 1 Department of Primary Care and Public Health, Cardiff University, School of Medicine, North Wales Clinical School, Gwenfro Units 6-7, Wrexham Technology Park, LL13 7YP, UK; 2 Centre for Health and Social Care Research, University of Huddersfield, UK; 3 Arthritis Research Campaign National Primary Care Centre, Keele University, UK; 4 South East Wales Trials Unit, School of Medicine, Cardiff University, UK; 5 School of Sports Health and Exercise Science, Bangor University, UK; 6 School of Nursing, Cardiff University, Cardiff, UK; 7 Centre for Economics and Policy in Health, Bangor University, UK; 8 North West Wales NHS Trust, Bangor, UK

## Abstract

**Background:**

Hip and knee osteoarthritis is a common cause of pain and disability, which can be improved by exercise interventions. However, regular exercise is uncommon in this group because the low physical activity level in the general population is probably reduced even further by pain related fear of movement. The best method of encouraging increased activity in this patient group is not known. A booklet has been developed for patients with hip or knee osteoarthritis. It focuses on changing disadvantageous beliefs and encouraging increased physical activity.

**Methods/Design:**

This paper describes the design of a Phase II randomised controlled trial (RCT) to test the effectiveness of this new booklet for patients with hip and knee osteoarthritis in influencing illness and treatment beliefs, and to assess the feasibility of conducting a larger definitive RCT in terms of health status and exercise behaviour. A computerised search of four general medical practice patients' record databases will identify patients older than 50 years of age who have consulted with hip or knee pain in the previous twelve months. A random sample of 120 will be invited to participate in the RCT comparing the new booklet with a control booklet, and we expect 100 to return final questionnaires. This trial will assess the feasibility of recruitment and randomisation, the suitability of the control intervention and outcome measurement tools, and will provide an estimate of effect size. Outcomes will include beliefs about hip and knee pain, beliefs about exercise, fear avoidance, level of physical activity, health status and health service costs. They will be measured at baseline, one month and three months.

**Discussion:**

We discuss the merits of testing effectiveness in a phase II trial, in terms of intermediate outcome measures, whilst testing the processes for a larger definitive trial. We also discuss the advantages and disadvantages of testing the psychometric properties of the primary outcome measures concurrently with the trial.

**Trial registration:**

Current Controlled Trials ISRCTN24554946

## Background

Systematic reviews have highlighted the effectiveness of exercise in reducing pain and disability in hip and knee osteoarthritis [1-4] and recent guidelines have emphasised the central role of exercise in the management of osteoarthritis [5,6]. Both aerobic walking and muscle strengthening exercise have been shown to be effective; however the optimal type, dose and setting for such physical activity is uncertain [3,7]. Despite these benefits, long-term adherence to exercise regimes is disappointing, and if exercise is not maintained its beneficial effects decline over time and finally disappear [8]. The level of physical activity in older adults in the United Kingdom is low [9-12], and reduced further by pain related fear of movement in those with osteoarthritis [13,14]. Indeed, there is a culturally conditioned response to pain that encourages rest, which is inappropriate for most with this problem. How can osteoarthritis patients be encouraged to increase their physical activity? In the similar field of low back pain an evidence-based patient advice booklet ('The Back Book' [15]) designed to encourage activity was found to be effective in changing patients' beliefs and behaviours. The effectiveness of this 'Back Book' was demonstrated in three randomised controlled trials (RCTs) [16-18], one of which involved older people.

### Developing 'The Hip and Knee Book'

We have developed a similar booklet encouraging increased activity in patients with hip or knee osteoarthritis. The theoretical framework underpinning this new booklet is Leventhal's theory of self regulation, which states that our coping response to illness is governed by our beliefs about the nature of the illness: how well we understand the symptoms (its identity), its chronicity, its controllability, its cause, and the seriousness of its consequences [19]. Educational interventions should emphasise that control is possible and within individuals' capabilities. This model has been extended to include treatment beliefs, so that when considering an intervention patients weigh up the perceived benefit in health gain with the perceived cost in terms of pain, fear and expectation of exacerbating the condition [20]. In addition, social learning theory states that an individual's ability to perform an activity (self-efficacy) is crucial to behaviour change [21]. The evidence based messages for this booklet were obtained from a review of systematic reviews and evidence based management guidelines. These were then converted into patient centred messages and combined in the narrative of a draft booklet. This draft booklet was examined in four focus groups of patients with hip or knee osteoarthritis, which improved the phrasing and emphasis of the patient centred messages in the final booklet [22]. The development of this booklet has been described in more detail elsewhere [23]. This paper reports upon the design of a RCT to test the effectiveness of this new booklet for patients with hip and knee osteoarthritis in terms of influencing illness and treatment beliefs, and to assess the feasibility of conducting a larger definitive RCT to measure change in health status and exercise behaviour [24].

## Methods/Design

### Study design

This study has several overlapping aims. In order to test the effectiveness of the new 'Hip and Knee Book' for changing illness and treatment related beliefs a RCT design is employed using outcome measures designed to measure such beliefs. We also want to assess the feasibility of conducting a future definitive RCT and concurrent economic evaluation of the effectiveness and cost-effectiveness of this new booklet on health status and exercise behaviour, so we will use this RCT to pilot recruitment and retention of participants recruited from primary care with hip or knee osteoarthritis, measure changes in health status, levels of physical activity and public agency costs. The results will inform the sample size calculation for a future Phase III RCT. We have developed instruments measuring illness and treatment beliefs from outcome measures designed for different purposes, so we will use this RCT to concurrently test the validity and responsiveness of these adapted instruments.

### Recruitment

#### Practice and participant selection

**Figure 1:**
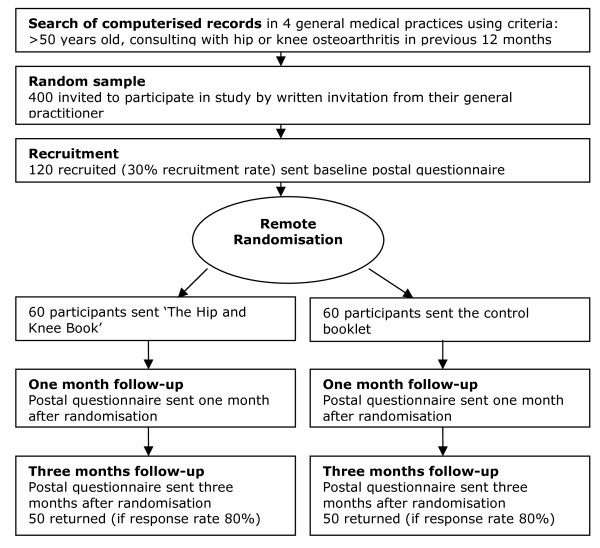
**Participant flow diagram**.

Participants will be recruited from four general medical practices in Wrexham and Flintshire in North East Wales in the United Kingdom. Patients with hip and knee osteoarthritis will be identified by searching the practices' computerised patient record database for the relevant Read codes (Table 1). Anonymised unique patient identification numbers will be downloaded onto an Excel file and sent to the trial statistician, who will select a random sample of 400 patients with hip and knee osteoarthritis from all four practices. Additional patients will be placed on a reserve list. The Excel file containing the anonymised patient identification numbers will be returned to the practice management team, who will check that the patients fulfil the inclusion and exclusion criteria and that it is appropriate to invite them to participate in the study. Patients who are ineligible, or where an invitation to participate would be inappropriate, will be replaced by those on the reserve list. The practice management team will send eligible patients invitation letters to participate, signed by their general practitioner (GP) on the practices' headed note paper, including an information sheet about the trial and a consent form. We will invite the initial 400 sample to participate, and those on the reserve list as necessary, until we have reached our target of 120 participants (Figure 1).

**Table 1:** Read codes for eligible patients (>50 years old)

**Included Read codes**	
N05 (and below)	osteoarthritis

14G2	H/O osteoarthritis

N06z5	hip arthritis

N06z6	knee arthritis

N094K	arthralgia of hip

N094M	arthralgia of knee

1M10	knee pain

**Excluded Read codes**	

M160	psoriatic arthropathy

N04 (and below)	inflammatory polyarthropathy

7K2 (and below)	hip joint operations

7K3 (and below)	knee joint operations

#### Inclusion and exclusion criteria

Patients over 50 years old presenting in primary care with hip or knee osteoarthritis within the last 12 months will be included. Specific diagnostic criteria for osteoarthritis will not be used as these are inconsistently applied in primary care populations. Exclusion criteria will be inflammatory joint disease, fractures, arthroplasty referral, and prescription of potent opioid analgesia. Patients who have already participated in the focus groups used to develop the new booklet will be excluded from the trial.

#### Informed consent

Patients will be sent an information sheet about the trial along with their letter of invitation and a consent form. The contact details of the research assistant co-ordinating recruitment will be included, and participants will be encouraged to ask for more information if they require it. The information sheet, letter of invitation and consent form will emphasise that patients' clinical care will remain unaffected whether they accept or decline to participate.

#### Registration and non-registration

Once the consent form has been returned to the trial manager (EA) at the trial office participant details will be recorded on an Access database and assigned a unique trial code. Anonymised details labelled with the unique trial code will be transferred to a separate database which will be used for recording all of the trial results. This will ensure that outcome measurement and statistical analysis will be performed blind to treatment allocation. All databases will be password protected. Eligible patients who are not part of the random sample will not be contacted and will not be invited to participate. Patients who return consent forms but decline the invitation to participate will be recorded anonymously. Completed consent forms will be stored in a locked filing cabinet.

#### Withdrawal & loss to follow-up

We anticipate that 100 out of the 120 (80%) participants recruited will return their final questionnaires. Participant withdrawal from the study will not affect their medical care, and this point will be emphasised in the patient information sheet.

#### Timing of randomisation

All units from a practice (block) will be recruited prior to randomisation. Randomisation will be undertaken by the trial statistician (JB) using information supplied on the baseline questionnaire.

### Study intervention

Participants randomised to the intervention arm will be sent the new educational booklet by mail. This booklet 'The Hip and Knee Book: helping you cope with osteoarthritis' [22] describes the development of osteoarthritis as a dynamic process with the potential for some repair and discourages the more passive concept of 'wear and tear'. Physical activity is emphasised as being beneficial for arthritic joints, as well as for general physical health, and a variety of different types of exercise are promoted. Patients are also encouraged to lose weight if overweight, use simple analgesia and anti-inflammatory gels. Prescribed medication and additional methods to control pain are described. The role of different therapists is discussed as are specialist treatments including arthroplasty. The booklet emphasises that control over their condition is possible and within individuals' capabilities.

Participants randomised to the control arm will be sent a patient information booklet about osteoarthritis produced by the Arthritis Research Campaign (ARC) [25], which can be obtained from the ARC website, and is widely available in rheumatology out-patient clinics. This booklet discusses the mechanism of osteoarthritis, treatment options and self-management. Physical activity and exercise is mentioned, but not emphasised. In particular, it does not address the same exercise-related beliefs and behaviours as the intervention booklet. Clinical care of patients will not be affected or influenced in any other manner by participating in the study.

### Outcome measures

#### Primary outcome measures

The primary outcomes will be illness and treatment beliefs. Illness beliefs will be measured with the Hip and Knee Beliefs Questionnaire (HKBQ). This has been modified from the Backs Beliefs Questionnaire (BBQ) [26], and measures beliefs regarding the progressive nature of hip and knee osteoarthritis. Treatment beliefs will be measured with a modified Exercise Attitude Questionnaire-18 (EAQ-18) [27]. We have demonstrated the face validity of these questionnaires in semi-structured interviews using cognitive de-briefing [28]. We will collect data on the reliability, validity and responsiveness of these modified questionnaires concurrently with the trial analysis. Using a transition question we will place those who report no change into a reliability analysis and those who do report change into a responsiveness analysis.

#### Secondary outcome measures

These will include:

• International Physical Activity Questionnaire (IPAQ) [29], a measure of physical activity over the previous seven days in terms of vigorous activities (heavy lifting, digging, aerobics, fast bicycling), moderate activities (carrying light loads, bicycling at a regular pace, doubles tennis), walking and sitting;

• Tampa Scale for Kinesiophobia (TSK) [Miller R, Kori S, Todd D. Unpublished report; 1991], a measure of fear-avoidance beliefs that has been used in osteoarthritis populations [30];

• Western Ontario McMaster universities Arthritis index (WOMAC) [31], a condition-specific outcome measure for osteoarthritis, which has sub-scales for pain, stiffness and disability;

• SF-12 [32], a generic outcome measure that provides a physical summary score and a mental summary score that can be compared across different illnesses and patient groups;

• EuroQol EQ-5D [33], a measure of health utility that can be used to calculate Quality Adjusted Life Years (QALYs) for a cost-utility analysis in an economic evaluation;

• Client Service Receipt Inventory (CSRI) [34,35], a measure of health service activity such as contact with health professionals in the community (general practitioners, nurse, health care assistants and others), tests or investigations, contact with secondary care health professionals (hospital doctors, specialist nurses and therapists), hospital services (accident and emergency, day surgery and in-patients) and medication, which can be combined with published national unit costs to measure costs for the economic evaluation;

• Adverse effects of the booklets collected as written comments at the end of the final questionnaire.

### Trial procedures

#### Postal questionnaires

The questionnaires will follow best practice in their design to maximise response rate [36]. In particular, the questionnaire design will be: aesthetically pleasing using coloured ink; as short as possible; accompanied by a personalised letter using the university letter head; and sent by first class mail. Non-responders will be contacted by telephone, and sent a second copy of the questionnaire if required. Postal questionnaires will be sent at baseline, after one and three months, in Teleform^® ^format to allow scanning of responses into a computerised database. All data will be anonymised and coded so that data collection and statistical analysis will be blinded to treatment allocation.

#### Health economic data collection

Health economic costs will be measured from a National Health Service (NHS) perspective. Health service activity will be collected from responses to the CSRI incorporated into the final patient questionnaire. Patients will be asked about the type and frequency of contact with primary and secondary healthcare services over the three month period. Unit costs will be obtained from national sources [37] and local finance officers.

### Randomisation

Randomisation will balance the two groups by site of pain, age, gender and duration of symptoms. As all patients are consented prior to randomisation, this will use an optimal allocation approach proposed by Raab [38] and algorithms developed by Carter [39]. Patients will be recruited from each practice and then randomised as a block. Within the block all potential allocations to two groups will be calculated and their balance statistic calculated. The 1% with the nearest optimal balance will be passed to an independent statistician who will randomly select the allocation for use and randomly allocate groups. The balance calculations for subsequent blocks will incorporate the degree of balance for the chosen allocation to ensure close to optimal balance across groups.

### Sample size

In order to detect a moderate to large effect size of 0.55 in change in illness and treatment beliefs, with 80% power and a significance level of 5%, a sample size of 100 will be needed. We anticipate that 30% of those invited will agree to participate, and 80% of those recruited will return their final questionnaires; so that 400 patients will need to be sent letters inviting them to participate, 120 will be recruited, and 100 will return final questionnaires.

### Trial analysis

All data will be anonymised and coded so that data collection and statistical analysis is blinded to treatment allocation. The code will only be broken after the main analysis has been completed.

#### Main analysis

Statistical analysis of the primary outcome measures HKBQ and EAQ-18 will be based on an intention to treat analysis using repeated measures analysis of covariance (ANCOVA). This method adjusts each patient's follow up score for his or her baseline measurements. Data transformations such as taking logarithms may be appropriate and any missing values can be imputed using regression techniques. The analysis will be expanded to include additional prognostic variables for exploratory analysis. There are assumptions underlying the ANCOVA model which include normality of residuals, equal variances, linearity, and independence. These assumptions will be tested, in particular the homogeneity of regression assumptions.

#### Analysis of secondary outcomes

Statistical analysis of the secondary outcome measures IPAQ, TSK, WOMAC, SF-12, EQ-5D will also be based on an intention to treat analysis using repeated measures ANCOVA. The effect size of WOMAC and IPAQ will inform the sample size calculation for a future phase III trial.

#### Assessment of feasibility of the phase III trial

Feasibility will be assessed by process measures of: the numbers of eligible participants identified, contacted and recruited with reasons for non-inclusion; the numbers of participants retained by the trial at one month and three months.

#### Psychometric testing of the modified outcome measures

The modified HKBQ and the modified EAQ-18 will be tested concurrently within the trial using criteria developed by Streiner and Norman [40].

Reliability will be measured in terms of internal consistency and test-retest reliability. Internal consistency will be measured using Cronbach's alpha on the baseline scores. This will be considered too low if below 0.7 and too high if greater than 0.9 to avoid item redundancy. Test-retest reliability will be assessed by comparing the baseline questionnaire with the retest questionnaires at one and three months. Transition questions to assess change in illness or treatment beliefs will be used with each follow up questionnaire. Those reporting that their beliefs were unchanged will be included in a test-retest analysis by calculating an intra-class correlation co-efficient (ICC) [41], and by calculating limits of agreement according to the method of Bland and Altman [42].

Construct validity will be assessed by comparing baseline with repeat questionnaires, if the transition questions indicate that beliefs have changed. Changes in beliefs will also be compared with changes in behaviour as measured by the physical activity questionnaire, and with changes in health status as measured by WOMAC and SF-12.

Responsiveness will be measured using the modified standardised response mean. The numerator of this statistic will be change in score when the transition question reports that beliefs have changed; the denominator will be the standard deviation of the change score when beliefs are unchanged [43].

### Cost effectiveness analysis

Mean health care costs of service use by patients in the control and intervention groups will be calculated and compared using non-parametric bootstrapping. Health economic analysis will initially involve a cost-consequences analysis of all the costs and outcomes collected [35]. We will calculate a bootstrapped incremental cost utility ratio (ICUR) point estimate of the cost per Quality Adjusted Life Year (QALY) to be gained from this intervention as compared with the control, using EQ-5D as a source of utility weights [33]. We will use bootstrapping to generate a cost-utility acceptability curve to inform policy makers of the probability that the intervention is cost-effective [35,44]. We will compare our estimate of the cost per QALY with the ceiling of £30,000 used by NICE. Given the relatively small sample size for this Phase II RCT, the findings will give a useful indication of the approximate size of the cost per QALY ratio for this intervention. It will inform the sample size calculation for a sufficiently powered future definitive RCT and concurrent economic evaluation. This trial platform offers an opportunity to compare EQ-5D values with SF-12 values and the other outcome measures of the trial in order to make some assessment of its sensitivity and appropriateness for use in a full trial [45].

### Ethical approval

Ethical approval was obtained from the North Wales East Research Ethics Committee [REC reference 09/WNo03/5].

## Discussion

A number of issues arise from our use of this phase II trial to not only test the processes for a larger definitive trial, but also to assess effectiveness in terms of intermediate outcomes and concurrently validate the primary outcome measures.

### Testing the effectiveness of intermediate outcomes

From our theoretical framework for developing 'The Hip and Knee Book' we have presumed that people with OA knee or hip have unhelpful beliefs and fears which are responsible for increasing their level of disability. We hypothesise that reading the new booklet, and not the control booklet, will alter their illness and exercise-related beliefs (measured by HKBQ and EAQ-18 respectively), reduce their fear of movement (measured by TSK), change their behaviour to increase their physical activity (measured by IPAQ), which will in turn reduce pain, stiffness and disability (measured by the condition-specific outcome measure WOMAC) and lead to improved general health status (measured by SF-12) and health utility (measured by EQ-5D). Outcomes measuring links higher up this theoretical chain are likely to be more responsive than links further down. Definitive or phase III trials need primary outcomes measuring health status or health utility, which are 'downstream', less responsive and require larger sample sizes. For this smaller phase II trial we have presumed that their will be a larger change in more 'upstream' intermediate outcomes such as illness and treatment-related beliefs. The position of these outcome measures in this theoretical chain can be debated. It may be that before illness and treatment beliefs change participants' fear of movement must be addressed. Alternatively change in participants' views about the prognosis of their arthritic condition, and the benefit of exercise, may lead to a reduction in the fear of movement. Self-efficacy is also important in changing behaviour such as exercise. Although we did not include a separate measure of self-efficacy so as not to over-burden our participants, some of the individual items in the EAQ-18 outcome measure do address this.

### Assessing feasibility for a larger phase III trial

According to the Medical Research Council's guidance for developing and evaluating complex interventions [24] the piloting stage includes: testing procedures for delivering the intervention, estimating the likely rates of recruitment and retention of participants, and estimating the effect size of the primary outcome measure in preparation for a sample size calculation. We have also taken the opportunity of testing outcomes measuring beliefs which we hypothesise will have a larger effect size than outcomes measuring behaviours or health status.

### Concurrent psychometric testing of outcome measures

The HKBQ has been adapted from the Back Beliefs Questionnaire [BBQ], which is a measure of patients' beliefs about the inevitable consequences of back pain, and was shown to be responsive to change in a previous RCT of 'The Back Book' [46]. It has also been adapted to become the Whiplash Beliefs Questionnaire, which has undergone psychometric testing [47]. A similar instrument for hip and knee OA is not available, so we decided to adapt the BBQ for this purpose. We believe that the risk that such an adapted outcome measure will not be reliable, valid or responsive is less than that if it were to be constructed *de novo*. Whilst the ideal situation would be to test its psychometric properties for this different population before using it in a trial; we took the pragmatic decision to validate it concurrently with the phase II trial. We have used this approach successfully in other previous studies [47,48]. The questionnaire is being administered with an intervention, which we hope will alter the outcome measures being tested, so the conditions are not ideal for assessing test-retest reliability. However, we will attempt to obtain some evidence about reliability by collecting data on those participants who claim that there is no change according to an accompanying transition question. Of course patients' responses to these outcome measures may shift independently of any trial intervention, in response to behavioural, cognitive and affective processes necessary to accommodate any chronic illness [49]. So long as these processes influence the outcome measures and transition question in a consistent manner, they should not interfere with the concurrent psychometric testing.

## List of abbreviations used

ANCOVA: Analysis of Co-variance; ARC: Arthritis Research Campaign; BBQ: Back Beliefs Questionnaire; CSRI: Client Service Receipt Inventory; EAQ-18: Exercise Attitude Questionnaire-18; EQ-5D: EuroQol EQ-5D; GP: General Practitioner; HKBQ: Hip and Knee Beliefs Questionnaire; ICC: Intra-class Correlation Co-efficient; ICUR: Incremental Cost-Utility Ratio; IPAQ: International Physical Activity Questionnaire; NHS: National Health Service; NICE: National Institute for Health and Clinical Excellence; OA: Osteoarthritis; QALY: Quality Adjusted Life Years; RCT: Randomised Controlled Trial; REC: Research Ethics Committee; SF-12: Short Form-12; TSK: Tampa Scale for Kinesiophobia; WOMAC: Western Ontario McMaster universities Arthritis index.

## Competing interests

The authors previously developed and wrote 'The Hip and Knee Book' which has been published by The Stationery Office.

## Authors' contributions

The principal responsibility for study design was from NHW, KH, RTE, CW with further input from KB, RDN, MH, RL, JJ, GA, PB. NHW managed the project. EA was the trial manager. RTE designed the pilot economic evaluation. JB conducted sample size calculations. JB, KH, NHW designed the analysis plan, PB described the underlying psychological model. NHW drafted the manuscript integrating written contributions from AE, JB, RTE, PB, JJ, KH. All authors read and commented on drafts and approved the final manuscript.

## Pre-publication history

The pre-publication history for this paper can be accessed here:


